# Apoplectic Motation

**Published:** 1904-10-29

**Authors:** 


### APOPLECTIC MOTATION.

Under the above title Dr. William Browning[1] describes a symptom which he has observed in several cases of cerebral hæmorrhage, and which he believes to be very suggestive, if not conclusive, evidence that the hæmorrhage is continuing. The symptom is a peculiar form of unrest. The patient, who usually has an anxious expression, is constantly moving. The head is turned uneasily from side to side, the legs are drawn up and presently extended again, or the patient keeps aimlessly putting his hand to his face, or twisting himself about in bed. The movements, Dr. Browning thinks, are not of mental origin, but are due to irritation, although he has not found that they indicate the site of the lesion. They cease when the effusion of blood ceases or when an exit for the blood is made by trephining. The symptom is not present in cases of embolism or softening. As a help towards deciding the proper line of treatment and the immediate prognosis, Dr. Browning's "apoplectic motation" is likely to be of good service, if wider experience proves his observations to be correct. The question of advising surgical interference in a case of cerebral hæmorrhage due to trauma, for example, is likely to be decided largely by an opinion as to whether the bleeding continues or has ceased; and such a comfortable way as Dr. Browning suggests of forming a judgment on that point would be indeed welcome.

1 Brooklyn Med. Jour., October 1904.

